# Same Day Identification and Full Panel Antimicrobial Susceptibility Testing of Bacteria from Positive Blood Culture Bottles Made Possible by a Combined Lysis-Filtration Method with MALDI-TOF VITEK Mass Spectrometry and the VITEK2 System

**DOI:** 10.1371/journal.pone.0087870

**Published:** 2014-02-14

**Authors:** Alexandra Machen, Tim Drake, Yun F. (Wayne) Wang

**Affiliations:** 1 Department of Pathology and laboratory Medicine, Emory University School of Medicine, Atlanta, Georgia, United States of America; 2 Department of Pathology and Laboratory Medicine, Grady Health System, Atlanta, Georgia, United States of America; University of Lausanne, Switzerland

## Abstract

Rapid identification and antimicrobial susceptibility testing of microorganisms causing bloodstream infections or sepsis have the potential to improve patient care. This proof-of-principle study evaluates the Lysis-Filtration Method for identification as well as antimicrobial susceptibility testing of bacteria directly from positive blood culture bottles in a clinical setting. A total of 100 non-duplicated positive blood cultures were tested and 1012 microorganism-antimicrobial combinations were assessed. An aliquot of non-charcoal blood culture broth was incubated with lysis buffer briefly before being filtered and washed. Microorganisms recovered from the filter membrane were first identified by using Matrix-Assisted Laser Desorption/Ionization Time-of-Flight VITEK® Mass Spectrometry (VITEK MS). After quick identification from VITEK MS, filtered microorganisms were inoculated to VITEK®2 system for full panel antimicrobial susceptibility testing analysis. Of 100 bottles tested, the VITEK MS resulted in 94.0% correct organism identification to the species level. Compared to the conventional antimicrobial susceptibility testing methods, direct antimicrobial susceptibility testing from VITEK®2 resulted in 93.5% (946/1012) category agreement of antimicrobials tested, with 3.6% (36/1012) minor error, 1.7% (7/1012) major error, and 1.3% (13/1012) very major error of antimicrobials. The average time to identification and antimicrobial susceptibility testing was 11.4 hours by using the Lysis-Filtration method for both VITEK MS and VITEK®2 compared to 56.3 hours by using conventional methods (p<0.00001). Thus, the same-day results of microorganism identification and antimicrobial susceptibility testing directly from positive blood culture can be achieved and can be used for appropriate antibiotic therapy and antibiotic stewardship.

## Introduction

Timely initiation of antimicrobial therapy is vital to the treatment of bloodstream infections and sepsis [1]–[7] which cause morbidity and mortality [1]–[5]. Significantly reducing the time to microbial identification (ID) and antimicrobial susceptibility testing (AST) could decrease the average time to appropriate antimicrobial therapy leading to a decrease in mortality, shortened hospital stay, and lower hospitalization costs [3]–[9].

The current standard for detecting microorganisms responsible for bloodstream infections and sepsis is blood culture in commercial blood culture systems using liquid medium [1], [3], [6]. When an automated blood culture system is used, signal-positive culture bottles are taken out of the culture system and an aliquot of blood is subcultured onto solid culture media and incubated until colonies are visible prior to species level ID [1], [3], [6]. Gram staining from positive blood culture provides only partial or certain information which can help guide empiric treatment plans [6]. Conventional ID includes growth and biochemical characteristics from culture media, or generated by commercial ID systems, which will take up to two days for many microorganisms and longer for others such as yeasts [3], [6]–[10]. Molecular methods such as DNA microarrays, fluorescent in-situ hybridization, and real-time polymerase chain reaction (RT-PCR) have shown to be efficient for the identification of specific microorganism. However, molecular methods lack the ability to identify a broad range of microorganisms and antimicrobial resistance [6], [8].

Matrix-Assisted Laser Desorption/Ionization Time-of-Flight Mass Spectrometry (MALDI-TOF MS) technology has been introduced as a way to quickly and accurately identify bacteria and yeast [4], [7]–[11]. Compared to standard phenotypic identification, this technology is rapid, inexpensive (after initial purchase of the instrument), and can identify bacteria grown on solid media to the species level [6], [9]–[10], [12]. We previously published a study describing a Lysis-Filtration Method (LFM) that can be used for ID of bacteria and yeast directly from positive BacT/ALERT® blood culture [6]. In this study, we asked if this method could be used for accurate full panel AST in a clinical setting. Full panel AST means susceptibility testing for multiple antimicrobials using minimum inhibitory concentration (MIC) which can be generated by commercial panels such as VITEK®2 cards on VITEK®2 system.

This proof-of-principle study aims to (1) utilize the Lysis-Filtration Method not only for identification by MALDI-TOF VITEK MS but also for direct antimicrobial susceptibility testing by VITEK® 2 AST system, (2) analyze the accuracy of this combined method, and (3) measure the turnaround time for ID as well as AST in a clinical setting.

## Materials and Methods

This study was approved by Emory IRB and was conducted in Grady Memorial Hospital, a 960-bed, inner city teaching hospital, during the period from 8/1/12 to 5/30/13. One hundred positive blood cultures from 100 different patients were included in the study. As there were no polymicrobic bottles seen during the study period, only monomicrobic isolates from positive blood bottles were studied.

Blood samples collected and inoculated in BacT/ALERT® anaerobic (SN) and standard aerobic (SA) non-charcoal based blood culture bottles, were incubated in the *BacT* **/** *ALERT* 3D system (bioMérieux, Durham, NC). When a signal-positive bottle was detected, aliquot was taken from positive bottles for Gram stain and subculture on solid media. Isolates grown from culture media were used for ID and full panel AST by using conventional methods such as MicroScan (Siemens, West Sacramento, CA) and VITEK® 2 (bioMérieux). In parallel, aliquot taken from positive blood culture bottles were processed using the LFM for direct ID by VITEK MS and full panel AST by VITEK2.

### Lysis-Filtration Method and Direct Identification by MALDI-TOF VITEK MS RUO

The Lysis-Filtration protocol for ID was previously published by our group [6]. Two milliliters of blood culture broth taken from either aerobic or anaerobic positive blood bottles were added to 1.0 ml of lysis buffer (0.6% polyoxyethylene 10 oleoyl ether [Brij 97] in 0.4 M [3-(cyclohexylamino)-1-propane sulfonic acid] [CAPS] filtered through a 0.2-µm-pore-size filter, pH 11.7), vortexed for 5 seconds, and allowed to incubate for 2 to 4 minutes at room temperature. The resulting lysate was filtered through a 25-mm 0.45-µm-pore-size filter (catalog no. HPWP02500; Millipore Express PLUS, Billerica, MA) for 40 seconds. The microbial cells remaining on the filter were washed three times with wash buffer (20 mM Na phosphate, 0.05% Brij 97, and 0.45% NaCl filtered through a 0.2-µm-pore-size filter, pH 7.2); washed three times with dionized water; and removed from the surface by scraping the filter with a micro-swab (Texwipe CleanTips swabs; catalog no. TX754B; Kernersville, NC). Sample processing time was approximately 10–15 minutes for up to three samples.

The MALDI-TOF VITEK MS RUO System with SARAMIS™ database by bioMérieux is a research use only (RUO) MS system. Microorganisms swabbed and recovered from filter paper were directly applied to VITEK MS target plates [6], and were covered with one microliter of CHCA matrix. If the VITEK MS was unable to identify an isolate on the first attempt, the sample was repeated using double the volume of blood culture broth and corresponding buffers. A bottle was considered to have a valid VITEK MS ID if at least one spot on the target slide gave a confidence level of ≥75% without conflicting identifications from replicate spots of the same sample. Bottles that did not generate an ID on the first attempt were repeated only once.

### Antimicrobial Susceptibility Testing by VITEK®2

Using the same micro-swab and membrane used for ID, a suspension was created following the Lysis-Filtration process and set aside until an ID result was obtained from the VITEK MS. As cellular debris is lyzed and filtered, bacteria left on the filter can be used for suspension preparation. The suspension was then adjusted to a McFarland standard of 0.5–0.63 for Gram positive and Gram negative bacteria and used for direct AST on VITEK®2 System. VITEK®2 cards were chosen according to the ID result given by the VITEK MS. In a few cases when an ID could not be obtained, Gram stain results were used to make choice of card, i.e., Gram positive AST card was used if Gram stain showed Gram-positive organisms. VITEK®2 cards were inoculated following manufacturers instruction. The ID from VITEK MS was introduced into the VITEK®2 system to allow it to choose the correct interpretive criteria. The resulting MIC was translated into clinical categories of susceptible, intermediate, or resistant following the Clinical and Laboratory Standards Institute (CLSI) recommendations.

Comparisons between the LFM and conventional methods were expressed as agreement, very major error (false susceptibility), major error (false resistance), or minor error (intermediate versus susceptible or resistant). This study used the clinical standard method of microdilution such as MicroScan and Etest as well as VITEK®2. The microdilution method by using MicroScan was the major method used as comparator with supplemental tests by Etest or VITEK2 if needed. The final ID and final AST results in the hospital clinical laboratory information system (LIS) were used for comparison.

### Statistical Analysis of Time

**Figure 1:**
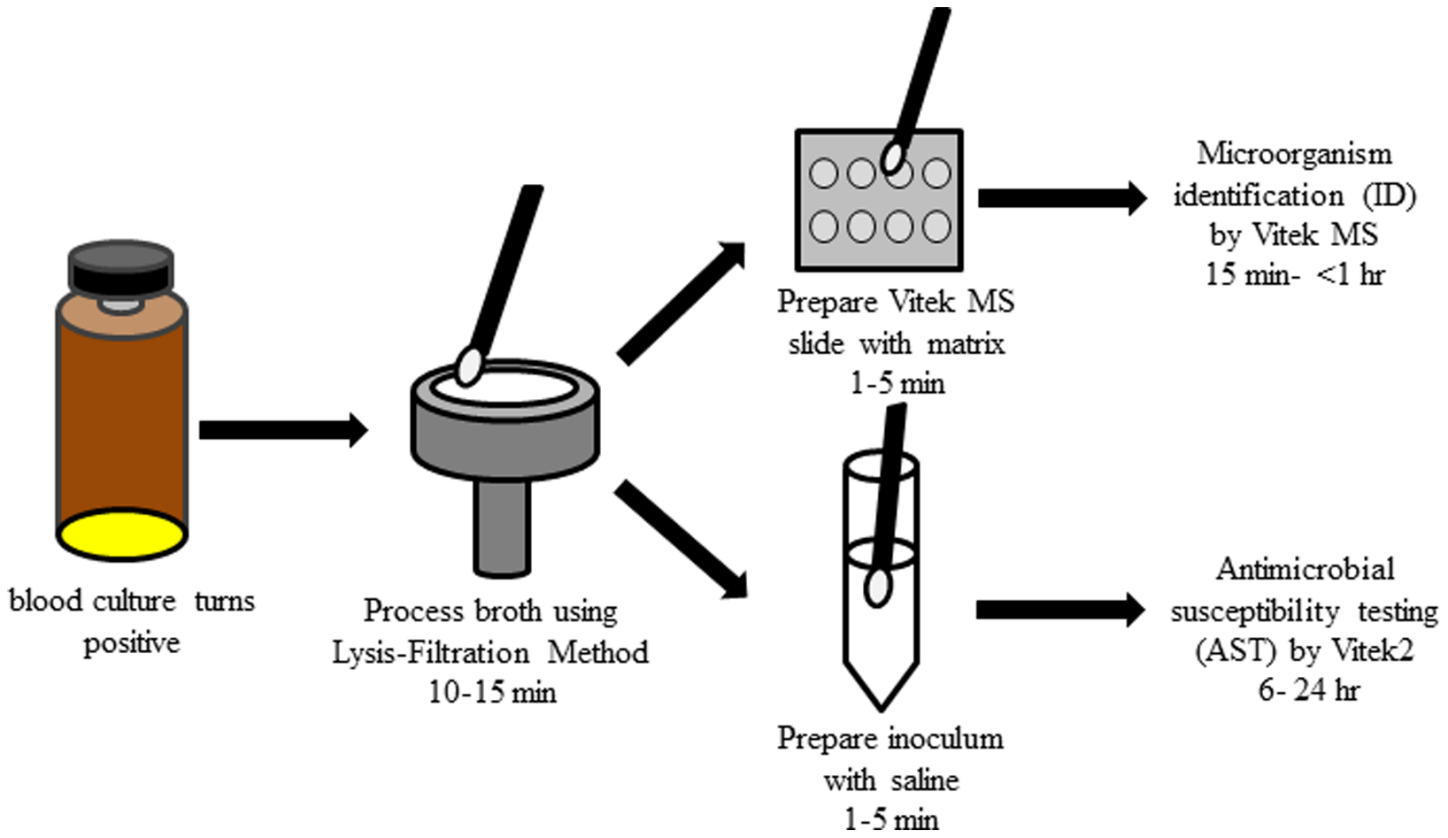
Process flow diagram for identification and antimicrobial susceptibility testing of bacteria directly from positive blood culture bottles using the Lysis-Filtration Method with MALDI-TOF VITEK Mass Spectrometry and the VITEK®2 System.

We compared the LFM to current standard practice in an effort to gain insight into how the process would improve clinical work flow. Figure 1 shows a process flow diagram of the LFM combined with VITEK MS and VITEK® 2. Student’s t-test was used for statistical comparisons of time. A p-value of <0.05 was considered statistically significant. All statistical tests were two-tailed.

## Results

### Direct Microorganism Identification

A total of 100 positive blood cultures from 100 patients were included in the study, consisting of 47 Gram positive, 49 Gram negative, and 4 yeast isolates. We compared our ID results to isolates grown from solid media culture and identified by conventional method such as MicroScan or VITEK®2. The VITEK MS identified 94.0% of isolates to the correct species level and 2.0% of isolates to the genus level (Table 1). The VITEK MS was unable to generate an ID for 3.0% of the isolates and incorrectly identified 1.0% of isolates. In detail, the VITEK MS was unable to identify one *C. glabrata* and one *S. aureus* isolate. One *K. pneumoniae* isolate was identified to the family level. One *Shigella* group C isolate was incorrectly identified by the VITEK MS as *E. coli*, which is a known limitation of MALDI-TOF MS.

**Table 1:** Susceptibility testing of bacteria from positive blood culture bottles by using a Lysis-Filtration Method, MALDI-TOF Mass Spectrum Analysis with SARAMIS Database, and the VITEK®2 System.

Organism	Number of isolates	Correct ID	Antimicrobials Tested	Agreement	Minor Error	Major Error	Very Major Error
**Gram-negative**	49	47	519	490 (94.4%)	21 (4.0%)	1 (0.2%)	7 (1.3%)
*Klebsiella pneumoniae* a	17	16	197	196	1	0	0
*Escherichia coli*	15	15	166	157	8	1	0
*Acinetobacter baumannii* b	4	4	32	28	4	0	0
*Citrobacter koseri*	1	1	11	11	0	0	0
*Enterobacter cloacae*	4	4	39	35	3	0	1
*Morganella morganii*	2	2	24	21	3	0	0
*Pseudomonas aeruginosa*	2	2	13	10	2	0	1
*Klebsiella oxytoca*	1	1	12	12	0	0	0
*Proteus mirabilis*	1	1	12	8	0	0	4
*Serratia marcescens*	1	1	11	10	0	0	1
*Shigella* group C^c^	1	0	2	2	0	0	0
**Gram-positive**	47	46	481	444 (92.3%)	15 (3.1%)	16 (3.3%)	6 (1.2%)
*Staphylococcus aureus* d	22	21	222	215	4	2	1
*Staphylococcus epidermidis*	14	14	164	137	10	12	5
*Staphylococcus capitis*	3	3	44	44	0	0	0
*Enterococcus faecalis*	2	2	8	8	0	0	0
*Enterococcus faecium*	2	2	8	5	1	2	0
*Streptococcus agalactiae*	2	2	3	3	0	0	0
*Staphylococcus haemolyticus*	1	1	16	16	0	0	0
*Staphylococcus hominis*	1	1	16	16	0	0	0
**Yeast**	4	3	12	12 (100%)	0 (0.0%)	0 (0.0%)	0 (0.0%)
*Candida albicans*	3	3	9	9	0	0	0
*Candida glabrata* d	1	0	3	3	0	0	0
**TOTAL**	100	96	1012	946 (93.5%)	36 (3.6%)	17 (1.7%)	13 (1.3%)

a One isolate was identified by VITEK MS to the family level.

b Two isolates were identified to the genus level.

c Identified by VITEK MS as *E. coli.*

d No ID by VITEK MS for one isolate.

### Direct Full Panel Antimicrobial Susceptibility Testing

For full panel AST testing, a total of 1012 microorganism-antimicrobial combinations were analyzed. The direct AST results from LFM for VITEK2 were compared to the conventional AST method reported in the clinical laboratory and documented in the LIS, which showed category agreement for 93.5% (946/1012) antimicrobials, minor error for 3.6% (36/1012) antimicrobials. Thus, 97.0% (982/1012) were either agreed or with minor error. There were results with major error for 1.7% (17/1012) antimicrobials, and very major error for 1.3% (13/1012) antimicrobials (Table 1). Microorganism-antimicrobial combinations that did not result in agreement with conventional methods are listed in Table 2. In detail, a small number of very major errors occurred in microorganisms such as *E. cloacae, P. mirabilis, P, aeruginosa, S. marcescens, S. aureus,* and *S. epidermidis* (Table 2).

**Table 2:** Microorganism-antimicrobial combinations that did not result in agreement with conventional methods^a^.

Microorganism	Minor Error	Major Error	Very Major Error
*Acinetobacter baumannii*	Ampicillin/Sulbactam, Levofloxacin	–	–
*Escherichia coli*	Ampicillin/Sulbactam, Cefazolin,Ceftazidime, Tobramycin	Cefazolin	–
*Enterobacter Cloacae*	Ceftriaxone, Tobramycin	–	Amikacin
*Enterococcus faecium*	Levofloxacin	Linezolid, Vancomycin	–
*Klebsiella pneumoniae*	Ampicillin/Sulbactam	–	–
*Morganella morganii*	Ciprofloxacin, Levofloxacin, Tobramycin	–	–
*Proteus mirabilis*	–	–	Ampicillin, Cefazolin, Ceftazidime, Ceftriaxone
*Pseudomonas aeruginosa*	Levofloxacin	Trimethoprim/Sulfamethoxazole	Ciprofloxacin
*Serratia marcescens*	–	–	Ceftazidime
*Staphylococcus aureus*	Levofloxacin, Clindamycin, Rifampicin, Vancomycin	Tetracyline, Vancomycin	Tetracycline
*Staphylococcus epidermidis*	Gentamicin, Clindamycin, Ciprofloxacin, Levofloxacin, Tetracyline	Clindamycin, Ertapenem, Rifampicin, Trimethoprim/Sulfamethoxazole, Vancomycin	Gentamicin, Trimethoprim/Sulfamethoxazole, Vancomycin

a Microorganisms not listed did not have any microorganism-antimicrobial combination errors.

### Time to Identification and Antimicrobial Susceptibility Testing

**Figure 2:**
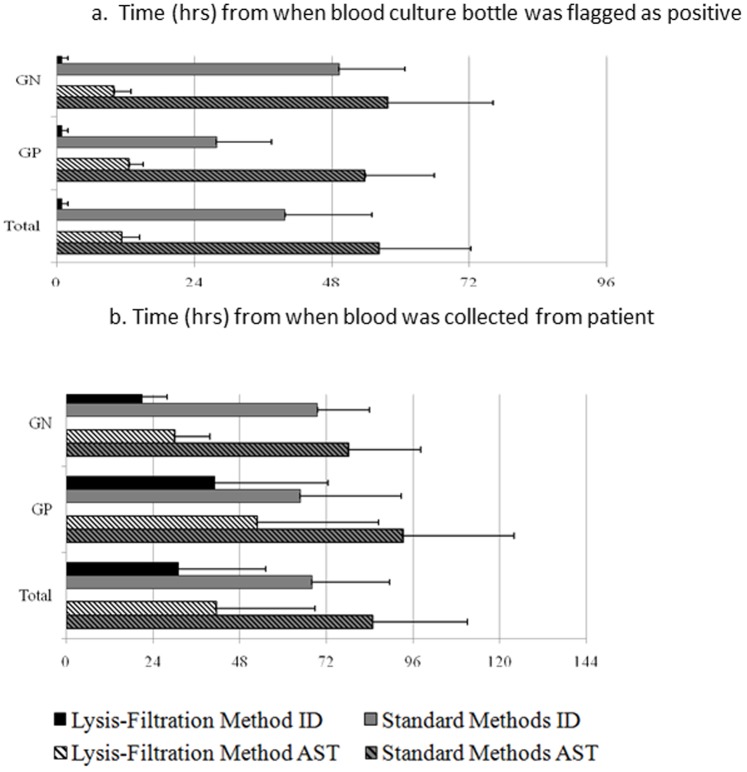
Average time in hours to results of ID or AST from (a) time when blood culture bottle was flagged as positive or (b) time when blood was collected from patient. For the Lysis-Filtration Method, ID was performed using VITEK MS, AST was performed using VITEK®2. AST = antimicrobial susceptibility testing. GN = Gram-negative organisms; GP = Gram-positive organisms; ID = identification; VITEK MS = MALDI-TOF VITEK MS RUO.

As shown in Figure 2 the time to microorganism ID and AST were analyzed by comparing time from blood sample collection and from the time blood culture bottle was flagged as positive.

Calculated from the time blood samples were collected in the blood culture bottles, the average time to ID was 31.1 hours by using combined LFM with VITEK MS as compared to 68.0 hours using conventional methods (p<0.00001). The average time to ID and AST from sample collection was 41.4 hours by using combined method vs. 84.8 hours using conventional methods (p<0.00001).

Due to variable times taken for blood cultures to turn positive, we analyze the turn-around times in another way, by calculating from the time a blood culture bottle was flagged as positive. The average time to organism ID was 1.0 hour using combined method as compared to 39.8 hours using conventional methods (p<0.00001). The average time from positive blood culture to ID and full panel AST result was 11.4 hours using combined LFM with VITEK MS and VITEK®2 vs. 56.3 hours using conventional methods (p<0.00001).

The median time from positive bottle to ID and AST was 9.9 hours using the combined LFM with VITEK MS and VITEK®2. In contrast, the median time to ID and AST was 54.7 hours by using conventional methods including subculture, ID, and AST from subculture using MicroScan, VITEK® 2, and/or Etest. For Gram negative isolates, time to ID and AST ranged from 6.0 to 16.8 hours by using the combined LFM, VITEK MS, and VITEK®2 methods, vs. 36.0 to 132.0 hours by using conventional methods. For Gram positive isolates, time to ID and AST ranged from 8.3 to 18.5 hours by using the combined method vs. 35.5 to 84.1 hours by conventional methods (Table 3).

**Table 3:** Comparison of time to ID and AST between Lysis-Filtration method and conventional methods (hours)^a^.

	Average		Median		Range	
Microorganism	Conventional Methods	Lysis-Filtration Method	Conventional Methods	Lysis-Filtration Method	Conventional Methods	Lysis-Filtration Method
Gram-negative	57.8	10.1	53.9	8.5	36.0–132.0	6.0–16.8
Gram-positive	53.9	12.7	54.8	10.5	35.5–84.1	8.3–18.5
Yeast	54.9	15.8	58.7	14.0	40.5–61.8	12.8–14.8
TOTAL	56.3	11.4	54.7	9.9	35.5–132.0	6.0–18.5

a Time was measured from when a blood culture bottle turned positive to ID and AST was reported.

Thus, the average times to ID and AST using the combined LFM with VITEK MS and VITEK2 were significantly shorter than those using conventional methods, calculated either from the time a blood sample was collected or the time a blood culture bottle turned positive.

## Discussion

In this study, we evaluated the Lysis-Filtration Method combined to VITEK MS and VITEK®2 for direct identification and direct antimicrobial susceptibility testing. Our combined method, compared to conventional methods, cuts down the time from sample collection to reported antimicrobial susceptibility by 43.4 hours for all microorganisms. One major advantage of our combined method is its simplified work flow. Microorganisms are transferred directly from the same filter to the VITEK MS and VITEK®2. Unlike conventional methods, it does not require Gram staining or subculture. Unlike centrifugation methods, it avoids extra steps as well as the potential of losing bacterial components during the removal of supernatant. The potential issue with this method would be remaining charcoal on the filter membrane if the clinical laboratory uses the charcoal containing blood culture bottles. We elected not to use the charcoal containing bottles.

In this study we evaluated the VITEK MS system instead of Bruker system. Previous studies that have focused solely on identification of microorganisms from blood culture have used a FLEX system by Bruker Daltonics (Bremen, Germany) [1]–[8], [10]–[11], [13]–[17]. A few studies have incorporated MS in the direct ID and AST of bacteremia, which used centrifuged pellets generated during the blood culture process and Bruker’s FLEX software for ID [1]–[2], [7], [18]. Wimmer *et al*. used centrifugation prior to ID and AST of Gram negative isolates in blood culture bottles [2]. Romero-Gomez *et al*. used a series of centrifugation, washes, and an extraction procedure before ID and AST [1]. The novelty of this study was the direct process from filtered blood samples to ID as well as full-panel AST which significantly improved the turn-around time of AST for organisms causing bloodstream infections.

The only incorrect ID was a *Shigella* group C isolate identified as an *E. coli. Shigella* species and *E. coli* have similar ribosomal protein sequences making it impossible for the current version of VITEK MS to differentiate from each other, which was reported previously [10], [19]. A few microorganisms of great clinical importance, 1 *C. glabrata*, 1 *S. aureus*, and 1 *K. pneumonia,* were not identified by the VITEK MS. The potential for cell debris remaining on the membrane might lead to incorrect identification or no identification by the VITEK MS. In previous studies [10], extraction methods have improved the performance of MALDI-TOF. In the future, adding an extraction step after lysis-filtration may improve results, specifically for yeasts.

MALDI-TOF MS has recently been applied to the detection of resistance with varying results. Vancomycin-resistance has been detected by MALDI-TOF MS in *E. faecium* isolates with the vanB gene. Methicillin-resistant *S. aureus* detection by MALDI-TOF MS is still a point of debate. Clinical *E. coli, K. pneumonia*, and *P. aeruginosa*, with B-lactamase resistance were not detected by MALDI-TOF MS. Unlike MALDI-TOF MS, VITEK®2 has a high sensitivity for AST and is highly standardized for AST [20]. We used VITEK®2 for full panel AST.

The percentage of major and very major error was low among the 1012 microorganism-antimicrobial combinations tested. *S. epidermidis* isolates, the coagulase negative staphylococci (CNS) that are common blood culture contaminants, exhibited the most errors among the Gram positive isolates. In the previous reports, CNS also had the highest rates of errors [1]. In both instances, this could be due to the high number of CNS isolates included in the study. Among the Gram negative isolates, the LFM performed similarly to the various centrifugation methods. Wimmer *et al*. reported the most errors for *E. coli* and *P. aeruginos* [2]. Romero-Gomez *et al*. study also saw very major error among *P. mirabilis* isolates [1]. With a category agreement of 93.5%, this method can be used to report preliminary AST results.

One limitation of this study is the limited number of yeast isolates included in the study. Yeast results were not singled out for statistical analysis, but were included in the total results as these organisms are an important part of the work flow process. Another limitation is that polymicrobial infections were not seen during the study period. Previous studies have reported difficulty in identifying polymicobic infections [6], [21]. Future larger studies should include more isolates, particularly more yeast isolates, as well as polymicrobial infections, to confirm our results.

The ultimate goal for using MALDI-TOF MS is to get early ID and AST for better use of antibiotics for treatment of infection [22]–[23]. To date, a handful of studies have investigated the clinical impact of MALDI-TOF MS on patient management and hospital costs [4]–[5], [7], [18]. Prompt identification by use of MALDI-TOF has been shown to cut down on time to appropriate antimicrobials [3]–[5]. This information can better guide antimicrobial management in settings with low levels of antimicrobial resistance [5]. In regions with high levels of antimicrobial resistance, rapid AST results are also needed. This proof-of-principle study provides a practical clinical method and tools for rapid ID and AST, which have the potential to impact antibiotic usage and clinical outcomes. Future studies are needed to assess this impact, as the time to ID or AST result is not the same as the time appropriate therapy.

This study demonstrates the effectiveness of the LFM combined with VITEK MS and VITEK® 2 for rapid and direct ID as well as AST of bacteria in a clinical setting: (1) the LFM produces a clean, concentrated sample of microorganism in less than 15 minutes; (2) the filtered sample can be used for direct ID by VITEK MS within an hour; and (3) the filtered sample can be used directly for full panel AST by VITEK®2 within 12 hours on average. Thus, same-day ID and full panel AST can be achieved in clinical setting with this easy to use method.

In summary, this study evaluated the Lysis-Filtration Method for rapid identification as well as direct full panel antimicrobial susceptibility testing of microorganisms directly from positive blood culture bottles in a clinical setting. This study indicates that results of microorganism identification and related antimicrobial susceptibility testing would be available for appropriate antibiotic therapy and antibiotic stewardship as early as the same day a blood culture turned positive.
